# Photoluminescence Switching in Quantum Dots Connected with Carboxylic Acid and Thiocarboxylic Acid End-Group Diarylethene Molecules

**DOI:** 10.1021/acs.jpcc.4c04978

**Published:** 2024-11-21

**Authors:** Ephraiem S. Sarabamoun, Pramod Aryal, Jonathan M. Bietsch, Maurice Curran, Sugandha Verma, Grayson Johnson, Lucy U. Yoon, Amelia G. Reid, Esther H. R. Tsai, Charles W. Machan, Christopher Paolucci, Guijun Wang, Joshua J. Choi

**Affiliations:** † Department of Physics, University of Virginia, Charlottesville, Virginia 22904, United States; ‡ Department of Chemistry and Biochemistry, Old Dominion University, Norfolk, Virginia 23529, United States; § Department of Chemistry, University of Virginia, PO Box 400319, Charlottesville, Virginia 22904, United States; ∥ Department of Chemical Engineering, University of Virginia, Charlottesville, Virginia 22904, United States; ⊥ Center for Functional Nanomaterials, Brookhaven National Laboratory, Upton, New York 11973, United States

## Abstract

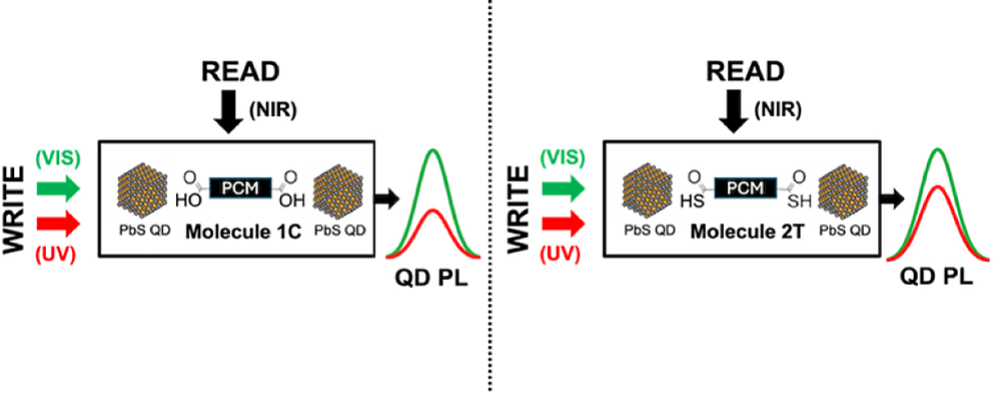

We contrast the switching of photoluminescence (PL) of PbS quantum dots (QDs) cross-linked with photochromic diarylethene molecules with different end groups, 4,4′-(1-cyclopentene-1,2-diyl)bis[5-methyl-2-thiophenecarboxylic acid] (**1C**) and 4,4′-(1-cyclopentene-1,2-diyl)bis[5-methyl-2-thiophenethiocarboxylic acid] (**2T**). Our results show that the QDs cross-linked with the carboxylic acid end group molecules (**1C**) exhibit a greater amount of switching in photoluminescence intensity compared to QDs cross-linked with the thiocarboxylic acid end group (**2T**). We also demonstrate that regardless of the molecule used, greater switching amounts are observed for smaller quantum dots. Varying these parameters allows for the fabrication of photoswitches with tunable PL change. We relate these observations to the differences in the HOMO energy levels between the QDs and the photochromic molecules. Our findings demonstrate how the size of the QDs and the energy levels of the linker ligands influences the charge tunneling rate and thus the PL switching performance in tunneling-based photoswitches.

## Introduction

In his landmark 1959 conference speech, Richard Feynman highlighted the enormous opportunities that lay in controlling and organizing matter on the smallest scales.^1^ One of the possibilities mentioned was the possibility of creating small structures that utilized quantum mechanical effects to produce novel material properties. In the decades since, the nanomaterials field has expanded enormously, and Feynman’s vision has come to fruition in nanoparticle systems like quantum dots. Quantum dots (QDs), for the purposes of this work, are nanoscale grains of semiconductors which utilize quantum confinement in three dimensions to produce materials that luminesce with high quantum yield and size-tunable optoelectronic characteristics. Due to these properties, QDs have been extensively studied for use in solar cells, LEDs, and biosensors.^2−10^ PbS quantum dots have been particularly interesting for near-infrared optoelectronics due to their convenient absorbance and emission ranges.^11^

As the QD research field matured, researchers have dramatically expanded their utility by pairing QDs with other interesting physical systems. Recently, the combination of photochromic molecules with QDs has emerged as a promising composite platform for different applications.^12−14^ photochromic molecules are molecules which exhibit reversible photoisomerization between two distinct geometric isomers. Such molecules have been studied for use in data storage/optical memory, bio imaging, and high-sensitivity optical switches.^15−20^ Among the different classes of photochromic molecules, diarylethenes have proven the most useful for many applications due to their good thermal stability, fatigue resistance, and ultrafast switching speeds.^21−23^ photochromic molecules have been used in conjunction with nanoparticles (NPs) like QDs for various applications including to create switchable catalysts, conductors, magnets, and even superconductors.^24−30^ For the purposes of this paper, however, we are primarily interested in the use of the QD/photochromic molecule composite system to produce a photoswitching effect. Such systems would leverage the high quantum efficiency of QDs and the rapid, reversible photoswitching of photochromic molecules to produce stable, promising photoswitches. A photoswitch is a device which can switch between two discrete states upon illumination and can be thought of as the optical counterpart of a transistor. These systems have been investigated both for use in optical computing (photonics)^31,32^ and in bioengineering as a minimally invasive biomarking and drug delivery system (photopharmacology).^33−36^

Historically, composite QD/photochromic molecule systems have utilized Förster Resonance Energy Transfer (FRET) between the QDs and the photochromic molecules as the photoswitching mechanism.^12,37−39^ While such photoswitches have been successful, their applicability is limited by the requirement to align the photochromic molecule absorbance with the photoluminescence (PL) of the QDs. Alternative mechanisms like Triplet Energy Transfer (TET) have also been employed,^40^ but again such mechanisms are limited by requiring the usage of smaller QDs with larger bandgaps. Previously, we have proposed a third mechanism for achieving photoswitching in this system by utilizing charge tunneling between photochromic molecule bridged QDs.^13,41^ In this process, the photochromic molecules bridging adjacent QDs effectively act as a tunneling barrier for excited QD charges. When the photochromic molecule changes its configuration, the tunneling rate is altered leading to a change in the overall QD PL of the sample. In this way, the well-known inter-QD charge tunneling effect, which has previously been studied for controlling optical properties^8,42^ and conductivity,^29,43,44^ in similar systems, is used to produce a photoswitching effect. Utilizing tunneling as the switching mechanism broadens the application range of this system and presents the only method for producing QD/photochromic molecule photoswitches that luminesce in the deeper infrared spectrum (1000–1500 nm). This allows for the fabrication of photoswitches with nondestructive readout which may be critical for certain applications, especially in photonics.

**Figure 1:**
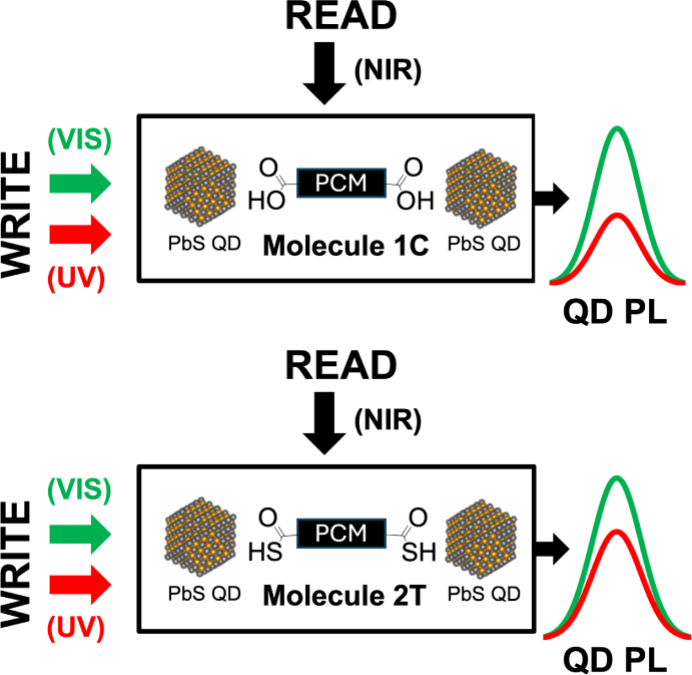
The key mechanism investigated in this work. UV and visible light are used to “write” or change the state of the system by changing the configuration of ligands **1C** and **2T** between their open and closed states. NIR light is used to “read” the state of the system. The difference in the binding group of the molecules results in a change in the observed QD PL switching.

In this work, we expand on our previous work to explore how varying the binding group of the bridging molecule can affect the PL switching effect of the photochromic molecule linked QDs. This is accomplished by contrasting the PL switching of the QDs linked by 4,4′-(1-cyclopentene-1,2-diyl)bis[5-methyl-2-thiophenecarboxylic acid] (**1C**) and 4,4′-(1-cyclopentene-1,2-diyl)bis[5-methyl-2-thiophenecarbothioic acid] (**2T**). Specifically, **1C** and **2T** are structurally identical except for their end groups: carboxylic acid for **1C** and thiocarboxylic acid for **2T**. By modifying the end groups of the bridging ligand, we hypothesized that differences in photochromic molecule energy levels and in the surface binding of the two ligands could alter the relative PL switching amounts of our system (Figure 1). Our work demonstrates that such a change in PL does reproducibly occur and provides some insights into the factors that might account for this change.

## Methods

### Syntheses and Ligand Exchange

#### PbS QDs Syntheses

Three PbS QD batches sized 3.4 nm [1], 4.3 nm [2], and 5.7 nm [3] were synthesized in the following way. Lead(II) oxide (3 mmol, 99.999%, Alfa Aesar) was mixed with 30 [1], 60 [2], and 90 [3] mmol of oleic acid (90%, Sigma-Aldrich) in a three-neck flask. An appropriate amount of 1-octadecene (90%, Alfa Aesar) was added to bring the total volume of the solution to 30 mL. The solution was stirred in the flask for 1 h under vacuum at 130 °C during which time it turned transparent. Under Argon gas flow, the temperature was adjusted to 130 °C for the first two syntheses [1–2] and to 95 °C for the last synthesis [3] in preparation for injection. In a nitrogen-filled glovebox, 0.1 M of hexamethyldisilathiane, (TMS)_2_S, solution was prepared by mixing 378 μL of (TMS)_2_S in 18 mL of 1-octadecene (ODE). Fifteen mL of the 0.1 M (TMS)_2_S solution was taken out of the glovebox and quickly injected into the three-neck flask. After 70 s, the reaction flask was submerged in an ice bath to quickly quench the temperature of the reaction solution. During the purification process, unreacted species were removed by a series of precipitation and centrifugation steps after cleaning the product with methyl acetate antisolvent and hexane solvent.

#### Ligand Exchange Mechanism by Deposition of Photochromic Molecules Dissolved in Methanol

A glass substrate was cleaned by consecutive rounds of sonication in soapy water (Hellmanex soap and deionized water), deionized water, isopropanol, and acetone. Slides were dried and treated with UV illumination for 5 min. 0.1 mL of a 10 mg/mL solution of QDs in tetrachloroethylene (TCE) was dispensed on the slide and the slide was spin-coated at 2000 rpm for 60s. Anhydrous methanol (0.1 mL) was deposited on the slide to minimize the effect of surface tension in preparation for photochromic molecule deposition. Next, a solution of 3.1 mM photochromic molecule in methanol (0.1 mL) was dispensed on the slide. This process was repeated twice more to ensure complete ligand exchange. After each application of the photochromic molecule solution, excess ligands were spin coated off the substrate at 2000 rpm for 60 s. After the exchange procedure, films were encapsulated with a 200-μm glass cover slide using epoxy curing. The slides were exposed to UV light for 20 min to allow the epoxy to harden, sealing the slide.

### Characterizations

#### Nuclear Magnetic Resonance (NMR) Measurements

Compound characterization was carried out through ^1^H NMR and proton-decoupled ^13^C NMR utilizing a Bruker 400 MHz NMR spectrometer in *d*_6_-DMSO or CDCl_3_. The CDCl_3_/DMSO-*d*_6_ peaks were calibrated at 7.26/2.50 ppm and at 77.0/39.5 ppm, in the ^1^H NMR and ^13^C NMR respectively.

#### Absorbance Measurements

**Figure 2:**
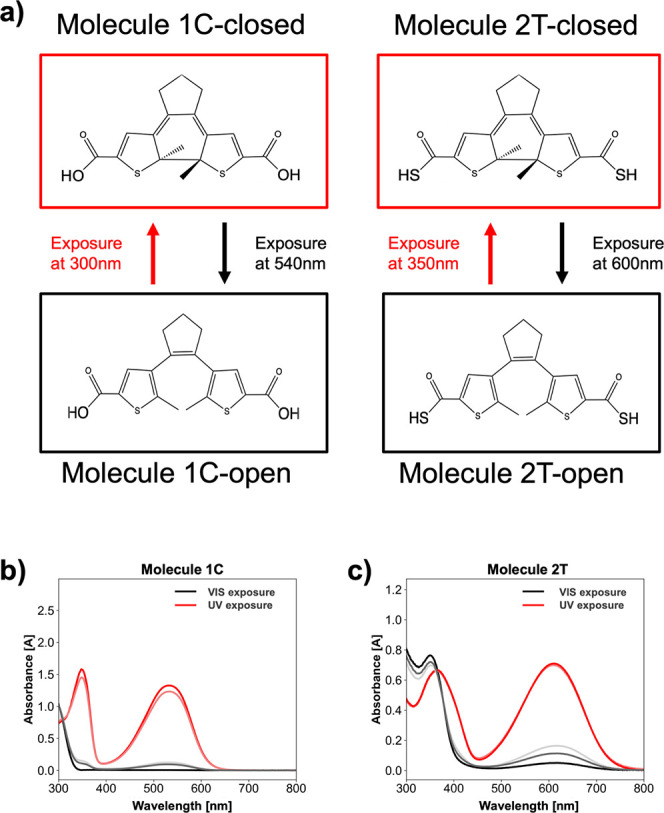
(a) Molecular structure of the “open” and “closed” configurations of **1C** and **2T**. Absorbance spectra of **(b) 1C** and **(c) 2T** in methanol after UV and visible light illumination. Darker lines denote longer light exposure, fainter lines denote shorter exposure times.

Absorbance measurements were performed using a PerkinElmer Lambda 950S spectrophotometer equipped with an integrating sphere. Ligands were dissolved in anhydrous methanol at concentrations of 11.7 mM in a 0.7 mL quartz cuvette. Path length of the absorbance measurement was estimated at 2.3 mm. UV and visible light exposures were performed using the same monochromatic light source utilized for PL measurements at the frequencies noted in Figure 2 of the manuscript (300 nm for UV exposure of **1C**, 540 nm for visible exposure of **1C**, 350 nm for UV exposure of **2T**, 600 nm for visible exposure of **2T**). Exposures were performed for up to 10 h to ensure maximal configuration.

#### Time-Resolved Photoluminescence (TR-PL) Measurements

Time-resolved PL was taken with a time correlated single photon counting setup with a pulsed 633 nm laser diode as the excitation light source. The spectrum of the laser diode was found to center at 636.5 nm with an fwhm of 4.7 nm. Time-resolved PL measurements were taken at the peak PL emission wavelength of the QD sample (1110 nm for the 3.4 nm QD sample). Measurement setup was identically repeated for slides interconnected with **1C** and **2T** ligands.

#### PL Measurements of the Photochromic Molecule Cross-Linked QD Thin Films

PL measurements were taken using a PTI Quantamaster 400 system. Quantum Dots were excited at 750 nm. The encapsulated photochromic molecule cross-linked QD thin film samples were placed on the stage in the spectrofluorometer. The light wavelength was changed to the appropriate wavelength to induce the configuration change of the photochromic molecules. Irradiation power was estimated as 0.33 mW for UV exposures and 0.070 mW for visible light exposure. Spot size of the sample was estimated as 8.35 × 10.0 mm. Exposures were performed for over 90 min per cycle to ensure maximal switching was achieved before measurements. Scans for measurement were conducted at a rate of 0.3 nm/s. After inducing the configuration change, without changing the position of the sample or any other parts of the setup, the excitation light wavelength was changed to 750 nm to obtain the PL spectra from QDs. All instrumental parameters such as slit widths, detector voltage, stage angle etc. were kept constant throughout the measurements.

#### GISAXS Measurements

GISAXS characterizations were performed at the 11-BM Complex Materials Scattering (CMS) beamline at the National Synchrotron Light Source II (NSLS-II) at Brookhaven National Laboratory. Thin film samples were measured at incident angles from 0.10 to 0.25° with a 200 μm (*H*) × 50 μm (*V*) beam at 13.5 keV (wavelength λ = 0.9184 Å). 2D scattering patterns were obtained using Dectris Pilatus 2M, 2 m downstream of the samples.

#### DPV Measurements

All DPV experiments were performed using a Metrohm Autolab PGSTAT302N potentiostat. The glassy carbon working (⌀ = 3 mm) and nonaqueous silver/silver chloride pseudoreference electrode behind a CoralPor frit were obtained from CH Instruments. The pseudoreference electrode was obtained by depositing chloride on bare silver wire in 10% HCl at oxidizing potentials and stored under light-free conditions in 0.1 M tetrabutylammonium hexafluorophosphate/acetonitrile solution prior to use. The counter electrode was a glassy carbon rod (⌀ = 3 mm). All experiments were performed in a modified scintillation vial (20 mL volume) as a single-chamber cell with a cap modified with ports for all electrodes and a sparging needle. Tetrabutylammonium hexafluorophosphate (TBAPF_6_) was purified by recrystallization from ethanol and dried in a vacuum oven before being stored in a desiccator. All data were referenced to an internal ferrocene standard (ferricenium/ferrocene reduction potential under stated conditions). All voltammograms were corrected for internal resistance during data collection. Experiments were carried out with a solution of 1.0 mM of molecules **1C** and **2T** in a 0.1 M TBAPF_6_/acetonitrile (MeCN) solution. The solution was saturated with argon before measurement for molecule **1C**. The experiments with molecule **2T** were done in a nitrogen atmosphere glovebox. Standard reduction potentials (*E*_1/2_) were determined from DPV utilizing the Parry-Osteryoung Equation^50^ where *E*_p_ is the peak potential and Δ*E* is the modulation amplitude:
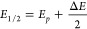


In all of these experiments, Δ*E* = 0.025 V, the modulation time is 0.01 s, the interval time is 0.1 s, and the scan rate is 50.354 mV/s,

### DFT Calculations

#### Calculated Absorbance, Energy Gap, and Orbital Diagrams for the Free Ligands

The Gaussian16 program was used with the hybrid functional B3LYP and the 6-311G basis set to generate the UV–visible spectra. The hybrid functional B3LYP was chosen since it was best suited to calculate the optical properties of our system.^51,52^ First, a ground-state geometry optimization was performed, followed by a frequency calculation to validate each minimum. A time dependent (TD)-DFT energy calculation was performed to compute the first three low-lying excited states. Following each TD-DFT energy calculation, the UV–vis absorbance spectra was generated and compared to experimental results. The calculated absorbance spectra were qualitatively consistent with experimental UV–vis spectra. The HOMO and LUMO energy values and orbital diagrams were recorded from the energy calculation outputs. Structures of the free ligands were obtained using the Chem3D modeling software. The geometrically optimized free ligand structures are included in the Supporting Information.

#### DOS for Adsorbed and Free Ligands

To simulate how the energy levels of the ligands may change when bound to the surface of the QDs, we calculated the HOMO/LUMO gaps for the adsorbed **1C** and **2T** groups on the PbS surface using the Vienna ab initio simulation package (VASP) version 5.4.4. The projector augmented wave (PAW) method^53^ was used and the PBE functional^54^ was used to describe the exchange-correlation potential. We used the D3-BJ dispersion corrections.^55^

To validate the use of the PBE functional, the HOMO/LUMO gaps for the free ligands in vacuum were first calculated by placing them in a 20 × 20 × 20 Å cell. We used a plane wave cutoff energy of 450 eV, the Brillouin zone was restricted to the Γ point, and we used the tetrahedron method with Blöchl corrections.^56^ The ligand geometries were first optimized, and geometries were considered converged when the energies and forces on each atom was less than 10^–8^ eV and 0.01 eV/Å, respectively. Next, the DOS were described as DOS = *f* (E–Ef), where E–Ef is the relative energy shift from the Fermi level (Ef). The DOS for the ligands obtained using the PBE functional were similar to those obtained for other diarylethene molecules in the literature,^57^ and the HOMO/LUMO gaps reproduced the Gaussian HOMO/LUMO gap results obtained earlier with a ∼ 1.55 eV offset (Table S2). GGA functionals, such as PBE, are well-known to underestimate HOMO/LUMO gaps compared to hybrid functionals (e.g., B3LYP), however, the GGA errors tend to be systematic and can be corrected by a constant energy offset.^58,59^ Using this offset, the PBE results were consistent with B3LYP. Thus, the PBE functional was used for further calculations on the QDs because combined with the offset it was reasonably accurate and is much less computationally expensive than B3LYP.

The (100) facet was used to model the quantum dot surface since this facet is thought to contribute most to the tunneling process.^60,61^ The (100) surface slab was simulated using a four-layer thick 3 × 2 periodic supercell and a vacuum spacing of 12 Å in the z direction to avoid interactions between surfaces. We used plane wave cutoff energy of 600 eV. The Brillouin zone was sampled using a Monkhorst–Pack *k*-point mesh, and the *k*-point sampling was set to 14 × 14 × 1 for slab structures. The ligands were adsorbed to the surface on one end and the geometries for the adsorbed ligands were optimized. The DOS were obtained for the adsorbed ligands as described above. This is an approximate model, since in the real material, ligands are adsorbed to QDs on both ends, and the exact bonding mechanism to the QD surface is disputed. This model does, however, preserve charge neutrality and gives a general idea of how the energy levels of the ligands might change in proximity to the QD surface. To isolate the energies of the ligands from the total DOS of the adsorbed ligands, the partial DOS was generated by summing up only the contributions of the ligand atoms and excluding contributions from the PbS. The structure files for all the geometries used in this work have been provided in the Supporting Information.

## Results and Discussion

Molecules **1C** and **2T** were synthesized and characterized to ensure purity (Figures S1 and S2).^62^ Ligands were dissolved in methanol for measurements and kept under inert atmosphere. Both molecules exhibited a reversible isomerization between “open” and “closed” states upon visible and UV illumination respectively (Figure 2a). The configuration change of the molecules was accompanied by a color change from pink to transparent for **1C** and from blue to transparent for **2T** (Figure S3). The pronounced color change is reflected in the absorbance spectra of the ligands (Figure 2b,c). Both ligands showed the pronounced formation of new absorbance bands in the visible regime after UV light exposure, and a corresponding suppression of those peaks after visible light exposure. For ligand **1C**, exposure at 300 nm resulted in bands centered around 350 and 540 nm. After exposure at 540 nm, the 350 and 540 nm bands were suppressed, and a new band formed around 300 nm. For ligand **2T**, 350 nm exposure resulted in band formations around 365 and 600 nm. After exposure at 600 nm, those absorbance bands were suppressed, and a new band formed around 350 nm. In addition to demonstrating the photochromic properties of these ligands, the absorbance results imply that both ligands experience near complete switching at least for the open configuration of the molecules as evidenced by the almost complete disappearance of the absorbance peak in the visible spectrum after visible light illumination.

To compose our photoswitches, three batches of different sized PbS QDs were synthesized and spin coated as thin films on a glass substrate. The absorbance, PL spectra, TEM images, and size-distribution histograms of the synthesized QDs are given in the Supporting Information (Figures S4 and S5). To exchange the native oleate ligands from the QD synthesis with the photochromic molecules, a solution of photochromic molecules in methanol was deposited and spin coated on the QD slides. Slides were exposed to monochromatic UV or visible light to switch the configuration of the photochromic molecule ligands to the “closed” and “open” states respectively, and PL measurements of the QDs were taken after each exposure. This was repeated three times with three consecutive cycles of UV and visible light exposure to demonstrate the reversibility of the photoswitch.

**Figure 3:**
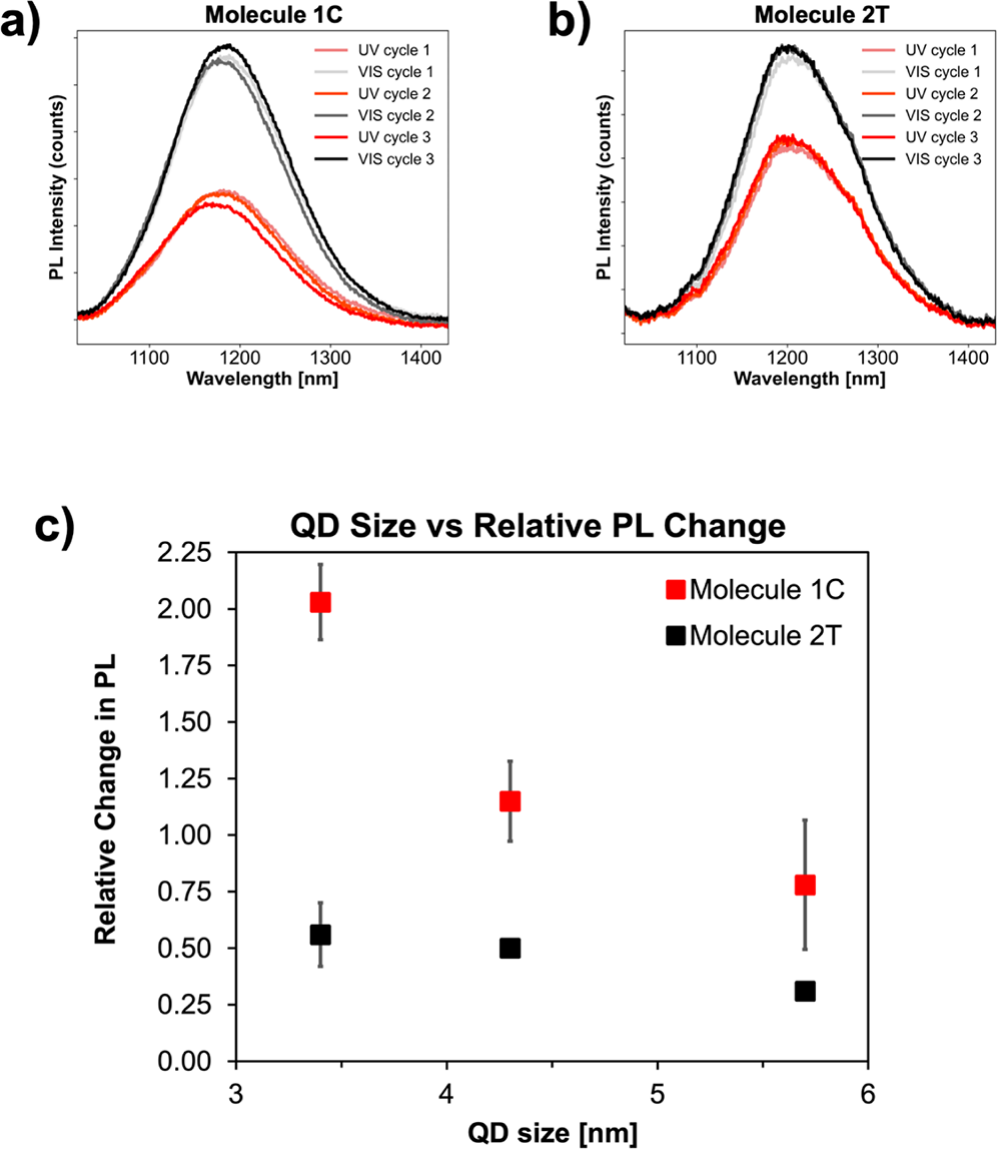
PL spectra of (**a**) **1C** and (**b**) **2T** cross-linked 4.3 nm QD films after successive cycles of UV and visible light illumination. (**c**) Measured switching amounts of three samples of QDs cross-linked with molecules **1C** or **2T.** The “Relative Change in PL” parameter is explicitly defined in eq 1 above.

Both **1C** and **2T** samples showed a robust and reversible photoswitching effect. Figure 3a,b shows the observed PL switching for the 4.3 nm QD sample cross-linked with molecules **1C** and **2T** respectively. The PL switching effect was found to be larger for the **1C** than the **2T** sample. To quantify the difference, we define A, the “relative change in PL” as follows:
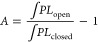
1where *PL*_*open*_ represents the PL curve of the “open” band, and *PL*_*closed*_ represents the PL curve of the “closed” band. Using this metric of eq 1, the average relative change in PL of the three cycles for **1C** is ∼1.20 whereas the average relative change in PL for **2T** is ∼0.50. These results for the 4.3 nm QDs were repeated with three slides to ensure consistency and experimental reliability (Figure S6). Results showed that **1C** samples consistently exhibited a significantly greater average relative change in PL than the **2T** samples. Subsequently, this procedure was repeated with different sized QDs (3.4, 4.3, and 5.7 nm). Raw data for these measurements are given in Figure S7. The average relative change in PL was plotted for both the **1C** and **2T** cross-linked slides as a function of the size of QDs used (Figure 3c). Results showed (1) a strong inverse correlation between the size of the QDs and the relative change in PL for both molecules **1C** and **2T**, and (2) that regardless of QD size, **1C** samples exhibited greater relative change in PL than **2T** samples. These results demonstrate that by varying the linker ligand and the size of the QDs, it is possible to tailor produce photoswitches to achieve desired switching magnitudes.

As described earlier, changing the configuration of the bridging photochromic molecule ligands is expected to change the charge tunneling rate between adjacent QDs. This would therefore lead to an expected difference in the overall excitonic lifetime in the different states of the photochromic molecules. To test this, time-resolved photoluminescence measurements were conducted on both the **1C** and **2T** samples in both their open and closed configurations (Figure S8). For **1C**, the excitonic lifetime was measured to be 59 ns in the open configuration and 33 ns in the closed configuration. For **2T**, lifetimes were measured to be 38 ns in the open configuration and 28 ns for the closed configuration. Both samples exhibited shorter excitonic lifetimes, and therefore higher inter-QD charge tunneling rates in the closed configuration of the photochromic molecules. These results line up well with the lower PL, suggesting a higher charge tunneling rate, of the closed configurations of both samples. Furthermore, the larger reduction in lifetime observed for **1C** compared to **2T** suggests a more dramatic change in the rate of tunneling for **1C** upon configuration change, which matches well with the greater observed photoswitching effect for **1C**.

Having established a difference in the **1C** and **2T** samples, we next aimed to analyze the possible causes of this difference. Our previous studies demonstrated that charge tunneling was the mechanism responsible for this photoswitching effect by carefully ruling out other possible mechanisms such as FRET and TET.^13,41^ We therefore proceeded to analyze our results in the context of the tunneling model. We examined three possible parameters which would affect the inter-QD tunneling rate and therefore result in the observed changes in the photoswitching effect of our samples. Those differences could include differences in “barrier width” (inter-QD distance), differences in the electronic coupling between the ligands and the surface of the QD, and differences in “barrier height” (energy levels of the photochromic molecules).

We first probed for possible differences in the “barrier width” of our **1C** and **2T** samples. This was accomplished by measuring the grazing incidence small-angle X-ray scattering (GISAXS) of the ligands to measure the inter-QD distances when the ligands are “open” and “closed” (Figure S9 and Table S1). Our results showed that the inter-QD distance remained ∼1 nm and varied by less than 1 Å for both the open and closed configurations of ligands **1C** and **2T**. This implies that the difference in “barrier width” was minimal between the two ligands and therefore is unlikely to account for the significant differences in photoswitching behavior. The GISAXS results also indirectly suggest that near complete ligand exchange of the photochromic molecules with the native oleate ligands was achieved for both **1C** and **2T** as evidenced by the short inter-QD distance measured for both samples.

**Figure 4:**
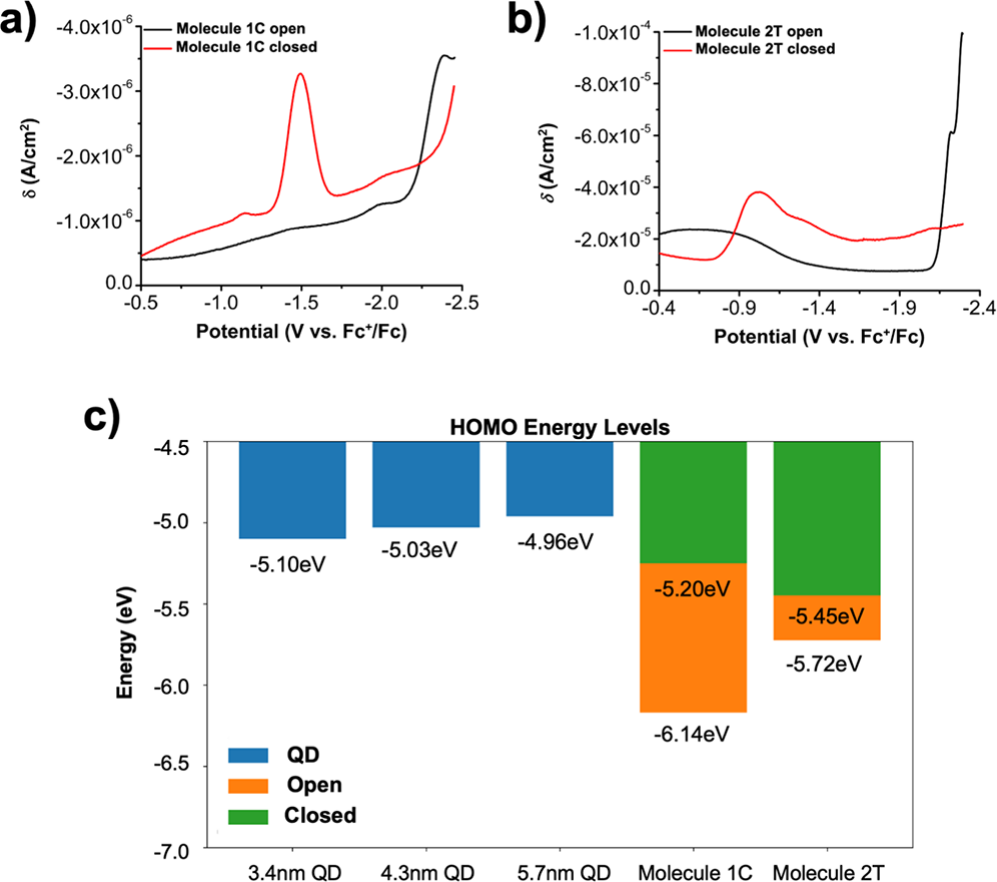
Reduction DPV plots for (a) **1C** and (b) **2T**. (c) HOMO energy level alignment of the system. QD valence band energies were obtained from literature^49^ and energies of **1C** and **2T** were determined from our DPV and absorbance results.

Second, we investigated possible differences in the electronic coupling of adjacent QDs through the different linker ligands. Generally, electronic coupling represents the amount of orbital overlap between an acceptor and a donor species.^45^ For our system, however, we are interested in the total electronic coupling between adjacent QDs through the linker ligand. To determine this factor, we must consider (1) the orbital structures of the photochromic molecules, and (2) the orbital overlap at the QD/photochromic molecule interface. The orbital structures of the photochromic molecules were assessed using DFT calculations. To ensure that our computational parameters correlate with our experimental results, the absorbances and HOMO/LUMO gaps of free **1C** and **2T** were calculated and compared to the experimentally obtained absorbance and HOMO/LUMO gaps. The calculated absorbance results (Figure S10) were qualitatively similar to the experimentally determined absorbance spectra of molecules **1C** and **2T** in both the open and closed configurations (Figure 2). Likewise, the calculated HOMO/LUMO gaps (Table S2) closely matched the experimentally obtained HOMO/LUMO gaps which are presented in Figure 4 below. Having established reasonable agreement with our experimental results, we next generated the electronic orbitals for both the HOMO and LUMO states of the “open” and “closed” configurations of both ligands. The orbital diagrams (Figure S11) show almost identical orbital structures for the two molecules with the exception of larger orbitals around the end-group sulfur atoms in **2T** than the corresponding oxygen atom in **1C**.

Since the orbital differences between the photochromic molecules are concentrated in the end groups, we focused on examining possible differences in orbital overlap of **1C** and **2T** at the QD/photochromic molecule interface. Here, the ligands likely deprotonate and a Pb–O or a Pb–S bond is formed between ligands **1C** or **2T** respectively with features on the QD surface.^46−48^ Given the similarity in the orbital structures of the ligands, we assumed that QD/photochromic molecule bonding is similar for the two ligands, and that differences in electronic coupling are mainly attributable to the difference in Pb–O versus Pb–S bond. Given the smaller disparity in electronegativity for the Pb–S bonds, greater electron sharing, and more orbital overlap is expected for the Pb–S bond compared to the Pb–O bond resulting in an enhanced electronic coupling for the **2T** ligand. This expectation does not match our PL results above and suggest that electronic coupling is likely not the dominant factor responsible for the differences in photoswitching between **1C** and **2T**.

Since neither differences in “barrier width” nor electronic coupling could explain why more switching was observed for the **1C** than the **2T** ligand, we moved on to investigate possible differences in the “barrier height” of our system. Given that the same batches of QDs were used for **1C** and **2T** slides, the difference in the “barrier height” must be due to differences in the energy levels of the two photochromic molecules. We therefore measured the energy levels of isolated **1C** and **2T** using differential pulse voltammetry (DPV) in acetonitrile. Focusing on the DPV reduction spectra of **1C** and **2T** (Figure 4a,b), a new peak was observed to form for both molecules after UV illumination. The first peaks in the reduction spectra were associated with the LUMO level of the molecules. For **1C**, an *E*_1/2_ of −1.48 V vs Fc^+^/Fc was observed which corresponds to a LUMO level of −2.92 eV. This value, taken with the molecular absorbance from Figure 2, gives a HOMO level of −5.2 eV for the closed state of **1C.** For the open state, **1C** has a peak at −2.38 V vs Fc^+^/Fc, which gives a LUMO level of −2.02 eV and a HOMO level of −6.14 eV. **1C** results match those that have appeared previously in literature.^41^ For **2T**, a redox wave appears around −1.02 V vs Fc^+^/Fc. This value corresponds to a LUMO level of −3.38 eV. This, taken with the absorption peak data, gives a HOMO level of −5.45 eV for the closed state of **2T**. For the open state, **2T** has its first reduction wave around −2.22 V vs Fc^+^/Fc, which corresponds to a LUMO level of −2.18 eV and a HOMO level of −5.72 eV. We note that the broad peak centered around −0.55 V vs Fc+/Fc for the open state of **2T** molecules is lower in energy than that of the closed state and thus is highly unlikely to correspond to the LUMO level of the open state **2T** molecules with a larger energy gap, as measured using absorption spectroscopy (Figure 2c). We speculate that the broad peak is caused by other processes such as adsorption of molecules to the electrode.

In our previous publications, we identified hole tunneling through the HOMO band as chiefly responsible for the observed tunneling effect.^13,41^ Therefore, special attention was given to the HOMO level alignment of our system. The HOMO energies from our DPV results for the ligands and the literature values for the HOMO energies of the quantum dots^49^ were used to graph the HOMO energy level alignment of our system (Figure 4c).

From our results, we clearly see a large difference in the measured energy levels of the two ligands. To analyze whether this difference in HOMO energy alignment may be responsible for the difference in the relative change in PL of the two ligands, we rely on the following equation which relates the two quantities:^41^
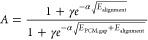
2

Here, A is the relative PL switching amount defined earlier in eq 1, γ is a dimensionless constant, α is a physical constant with units of *eV*^–1/2^, *E*_*alignment*_ = *E*_*PCM,closed*_*–E*_*QD*_ which represents the difference between the closed state photochromic molecule energy levels and the energy levels of the QDs, and *E*_*PCM,gap*_ = *E*_*PCM,open*_*–E*_*PCM,closed*_ which gives the energy level change between the “open” and “closed” configurations of the photochromic molecules. eq 2 implies that A is maximized when *E*_*alignment*_ is minimized and *E*_*PCM,gap*_ is maximized. Using our DPV results, *E*_*PCM,gap*_ = 0.94 eV for **1C** and *E*_*PCM,gap*_ = 0.27 eV for **2T**, and *E*_*alignment*_ ∼ 0.25 eV smaller for **1C** than it is for **2T**. Both these factors would result in a larger expected relative change in PL for molecule **1C** than for molecule **2T**. Therefore, given the relation in eq 2 the differences in the energy levels of the two ligands would lead us to expect greater relative PL switching for **1C** than for **2T**. Furthermore, comparing the energy levels of the different sized QDs, we note a smaller *E*_*alignment*_ for the smaller sized QDs which would lead us to expect a higher relative PL switching amount for smaller sized QDs. Therefore, the energy level results are consistent with our experimental observations depicted in Figure 3, suggesting that variations in energy levels may be the primary driver behind the differences in observed PL switching amounts.

**Figure 5:**
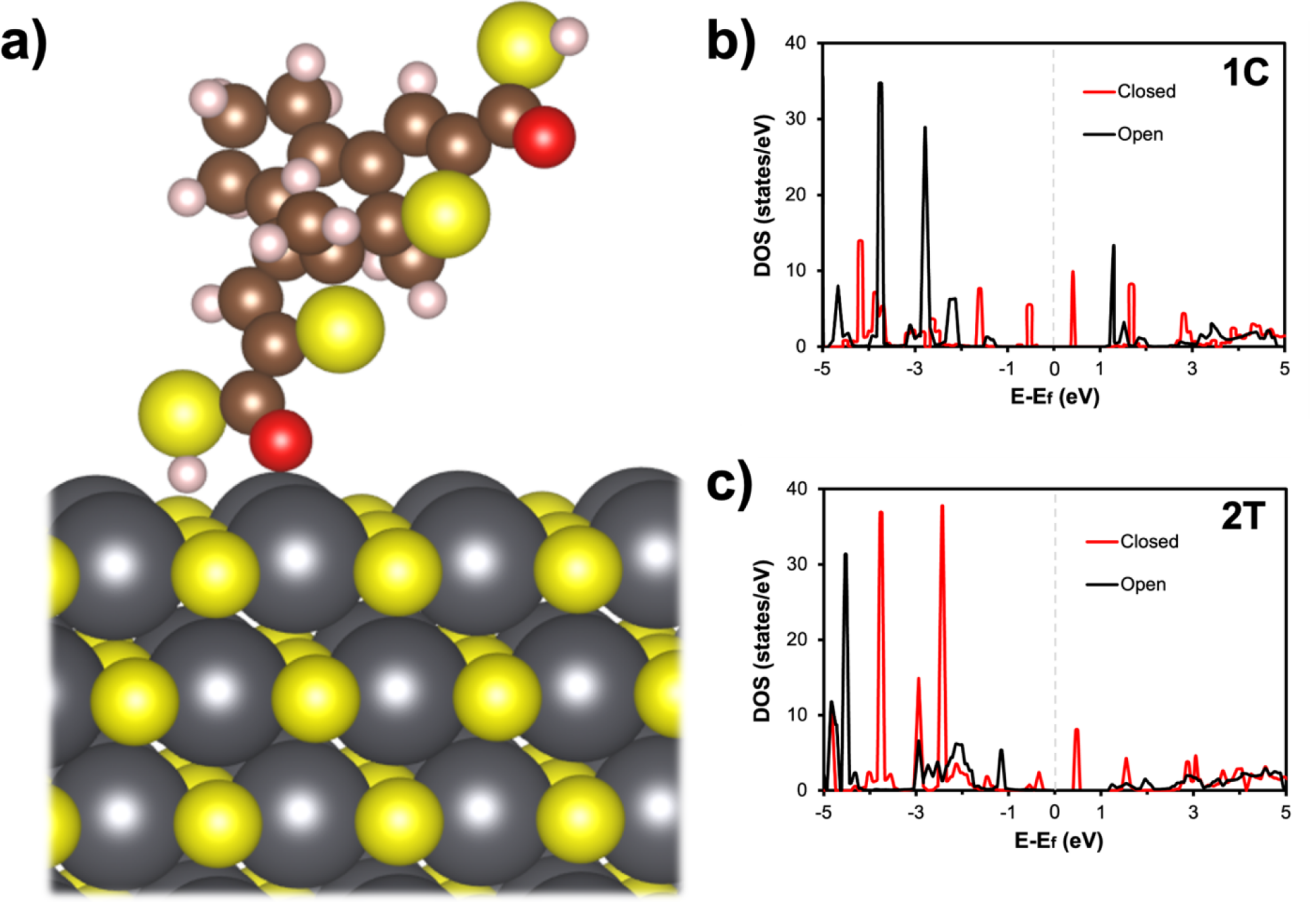
(a) Structure of the open **2T** ligand when adsorbed to the QD surface. Gray atoms represent lead, yellow atoms sulfur, brown atoms carbon, white atoms hydrogen, and red atoms oxygen. Calculated partial DOS for molecules (b) **1C** and (c) **2T** when adsorbed to the PbS surface (contributions from the Pb and S in the surface are excluded). Fermi energies are set to zero.

The measured DPV energy levels of the photochromic molecules above are only those of the unbound ligands, therefore, they do not account for any energy level shift(s) due to QD surface binding. Because the tunneling process is highly sensitive to the energy levels of the molecules, and strong QD absorbance in the visible regime makes absorbance measurements of the bound photochromic molecules impractical, DFT calculations were employed to investigate possible changes in the energy levels of the ligands when bound to the QDs (See experimental methods section for more details). To accomplish this, we generated a four-layer thick 3 × 2 (100) surface of PbS (Fm3̅m phase) to approximate the QD surface. We then placed and optimized the structure of the **1C** and **2T** ligands on the PbS surface (Figure 5a), and subsequently computed the density of states (DOS) of the open and closed conformers for both structures (Figure S12). To isolate the energy levels of the ligands, partial DOS for QD-bound **1C** (Figure 5b) and **2T** (Figure 5c) were generated by excluding contributions from the PbS surface. The partial DOS of the adsorbed ligands retain similar PBE-computed HOMO/LUMO gaps (Table S3) compared to the free ligands (Figure S13). Most importantly, the HOMO/LUMO gaps for the adsorbed ligands preserve their relative order, with molecule **1C** having larger open and closed HOMO/LUMO gaps than molecule **2T**. Although these results are based on a simplified model of our experimental system (see methods section for more details), they suggest that the energy alignment observed for the free ligands (Figure 4) is preserved when the ligands are bound to the QDs, suggesting that our analysis based on the measured DPV energies of the photochromic molecules remains valid.

Taken together, our DFT and DPV results suggest that modifying the end group of our photochromic molecules leads to changes both in the energy barrier and the electronic coupling between adjacent QDs. Both these factors are expected to affect the tunneling rate and therefore may contribute to the difference in the PL switching observed in **1C** and **2T**. Our analysis suggests that differences in the HOMO energy levels of our ligands is likely the primary driver behind the differences in PL switching observed, as this explanation better matches our experimental observations. We, therefore, recommend that future studies aiming to isolate and examine the effects of electronic coupling on the amount of PL switching in QD/photochromic molecule photoswitches should choose photochromic molecules with different end groups, but similar HOMO levels. This should minimize the impact of differences in energy levels on tunneling rate and allow for the careful study of the effect of electronic coupling on charge tunneling and PL switching.

## Conclusion

In this work, we investigated how changes in the end group of the photochromic molecule can affect the relative change in PL of tunneling based QD/photochromic molecule photoswitches. We demonstrated that molecule **1C** with carboxylic acid end groups has consistently and significantly more relative PL switching than molecule **2T** with thiocarboxylic acid end groups. We also demonstrated an inverse relationship between the size of the QD used and the photoswitching effect observed. Using GISAXS, DPV, and DFT calculations we explored the possible causes for the observed difference in switching behavior of the two ligands. Our findings show a possible connection between the differences in relative change in PL and the differences in the energy levels of the two photochromic molecules. This suggests that for the design of similar tunneling based QD/photochromic molecule photoswitches, special attention must be paid to the energy alignments between the ligands and the QDs. The careful selection of these energy relationships allows for the composition of photoswitches with customizable switching amounts.

## Supporting Information Available

^1^H and ^13^C NMR spectra of photochromic molecules **1C** and **2T**, PbS QD optical characterizations, raw PL spectra for **1C** and **2T** samples, GISAXS results of photochromic molecule bridged QD assemblies, alternative ligand exchange mechanism, and extra relevant DFT results (PDF)
Compressed file containing structures of the free ligands and adsorbed ligands used for DFT calculations (ZIP)
