# Supplementary material for: Photoluminescence Switching in Quantum Dots Connected with Carboxylic Acid and Thiocarboxylic Acid End-Group Diarylethene Molecules
Source: J Phys Chem C Nanomater Interfaces. 2024 Nov 21;128(48):20599–608. doi: 10.1021/acs.jpcc.4c04978 (PMC11626517; doi:10.1021/acs.jpcc.4c04978)
Supplement: Supplementary file 1 — jp4c04978_si_001.pdf [file jp4c04978_si_001.pdf]

Supporting Information

Photoluminescence Switching in Quantum Dots Connected with Carboxylic Acid and Thiocarboxylic Acid End-Group Diarylethene Molecules

Ephraiem S. Sarabamoun,[a] Pramod Aryal,[b] Jonathan M. Bietsch,[b] Maurice Curran,[d] Sugandha Verma, [d] Grayson Johnson,[d] Lucy U. Yoon, [d] Amelia G. Reid,[c] Esther H. R. Tsai,[e] Charles W. Machan,[c] Christopher Paolucci, [d] Guijun Wang,[*,b] Joshua J. Choi[*,d]
[a] Department of Physics, University of Virginia, Charlottesville, VA 22904, USA
[b] Department of Chemistry and Biochemistry, Old Dominion University, Norfolk, VA 23529, USA
[c] Department of Chemistry, University of Virginia, PO Box 400319, Charlottesville, VA 22904, USA
[d] Department of Chemical Engineering, University of Virginia, Charlottesville, VA 22904, USA
[e] Center for Functional Nanomaterials, Brookhaven National Laboratory, Upton, NY 11973, USA
* Corresponding authors
E-mails: jjc6z@virginia.edu, g1wang@odu.edu

Compounds **1C** and **2T** were synthesized via similar procedures to those in literature.[1-4] ^{1}H and ^{13}C NMR spectra of the synthesized compounds are provided:

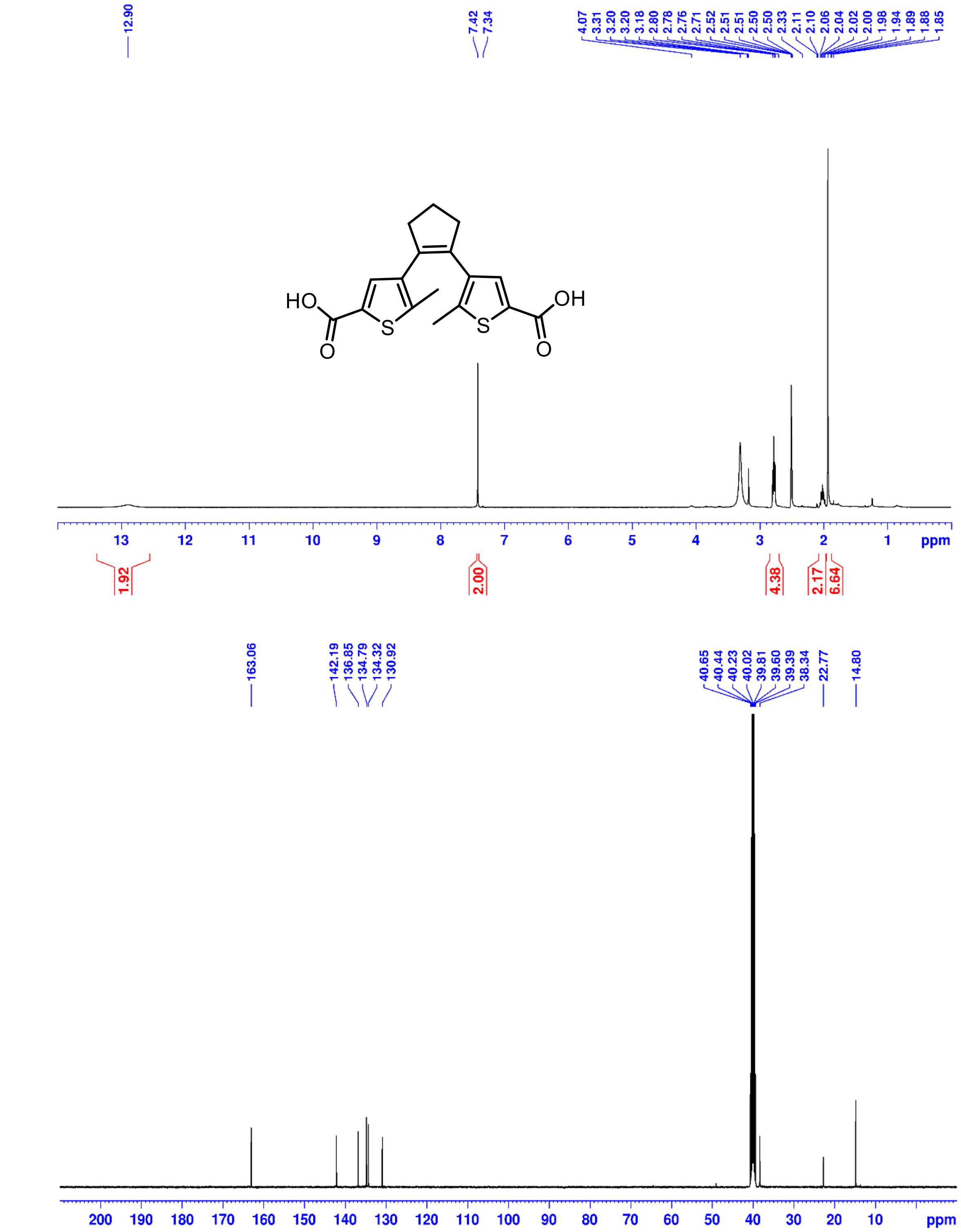

Figure S1. ^{1}H and ^{13}C NMR spectra of compound **1C** in D_6-DMSO, 400 MHz Bruker

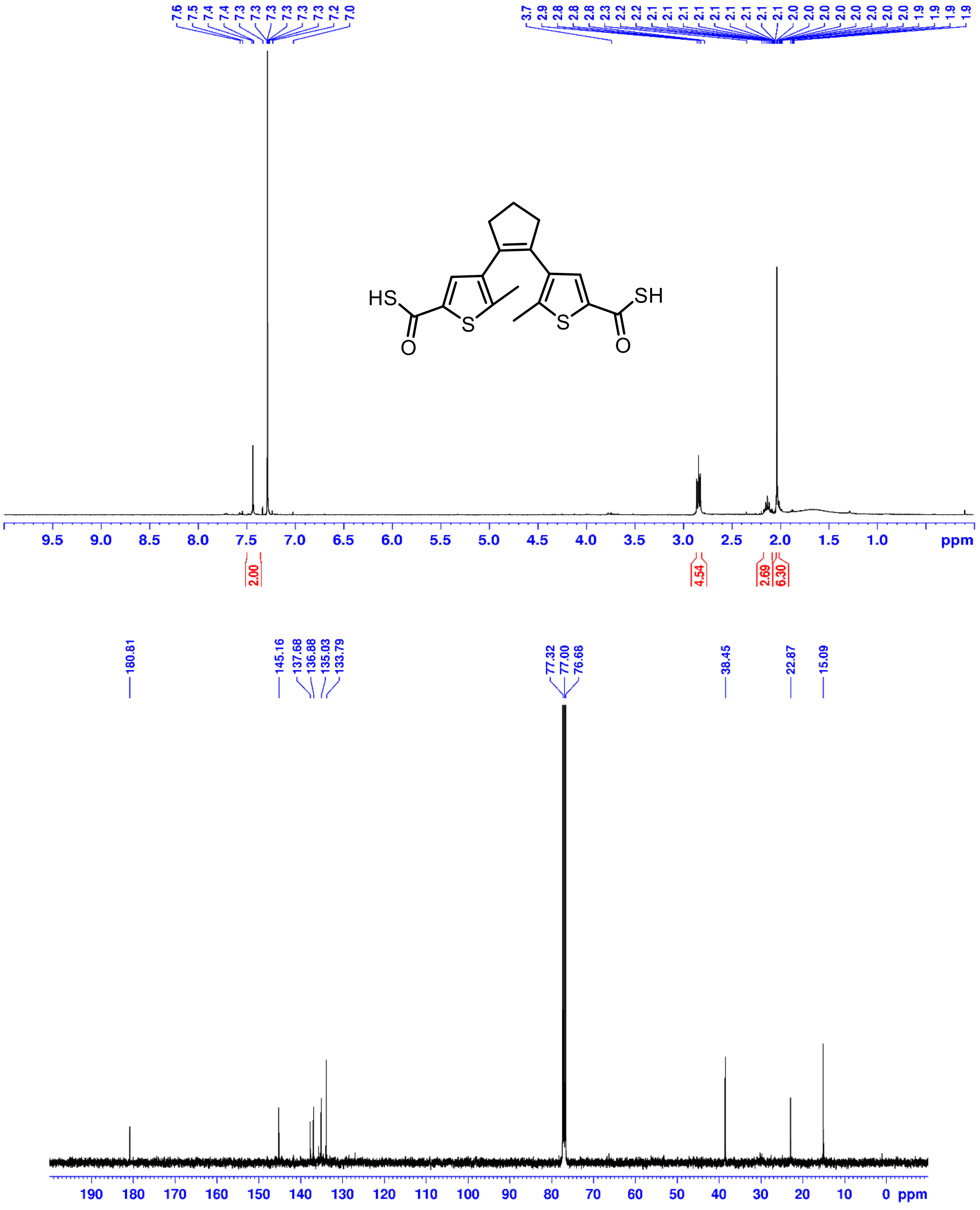

Figure S2. ^{1}H and ^{13}C NMR spectra of compound **2T** in $CDCl_3$, 400 MHz Bruker

Photochromic molecules in Methanol Color Change:

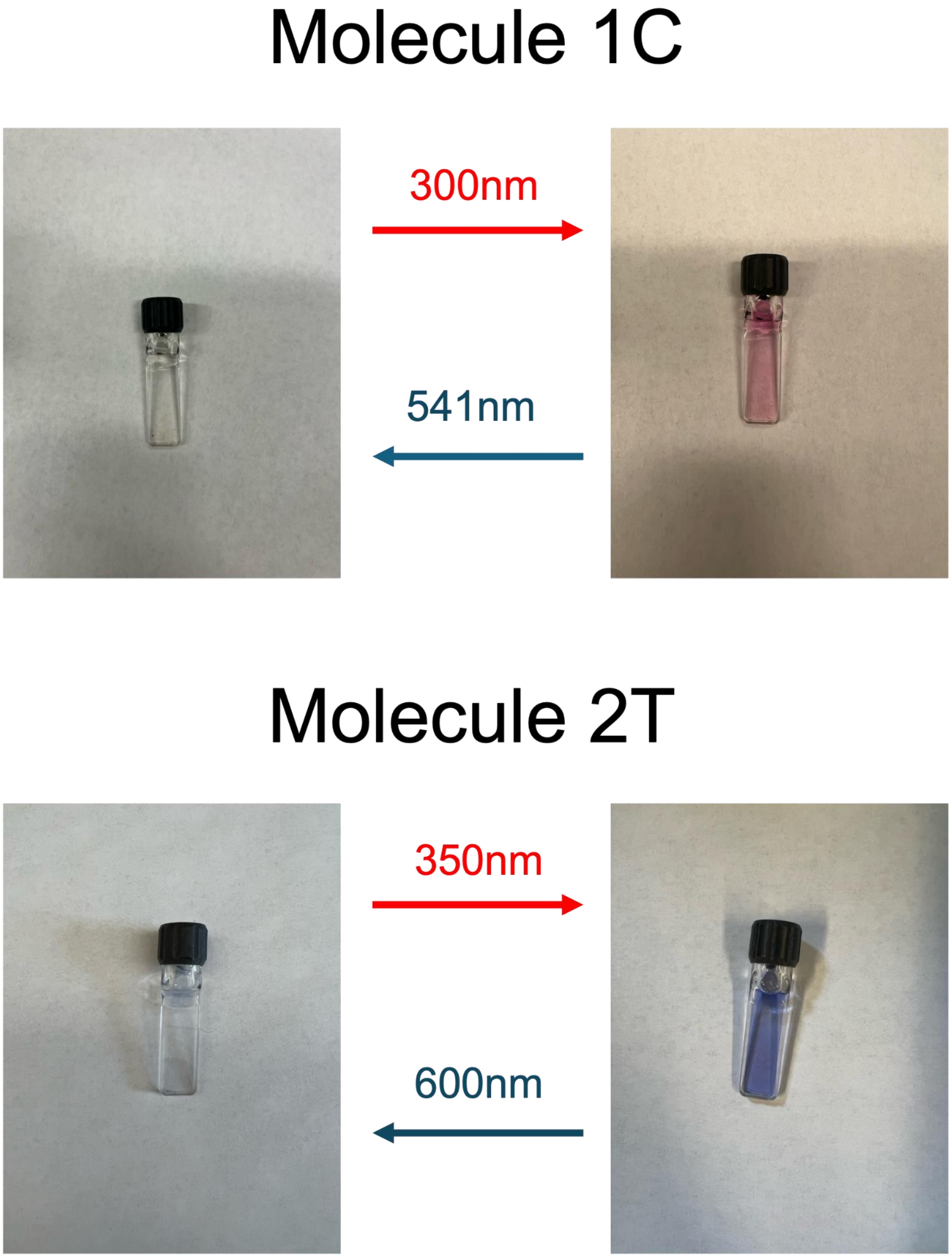

Figure S3. Ligands **1C** and **2T** both undergo noticeable color change upon change in configuration.

Synthesized QDs Absorbance, PL, and TEM Images:

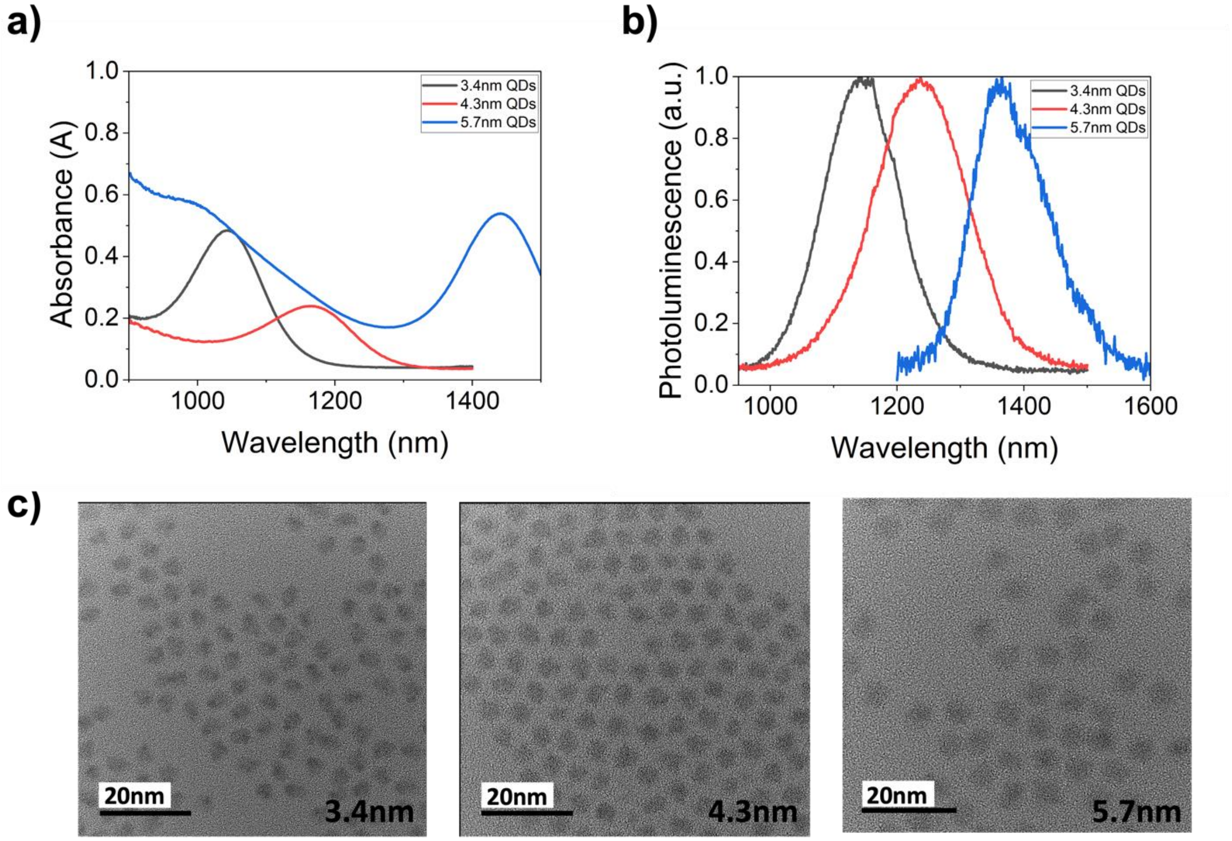

Figure S4. (**a**) Absorbance, (**b**) PL spectra, and **(c)** TEM images of the synthesized QDs

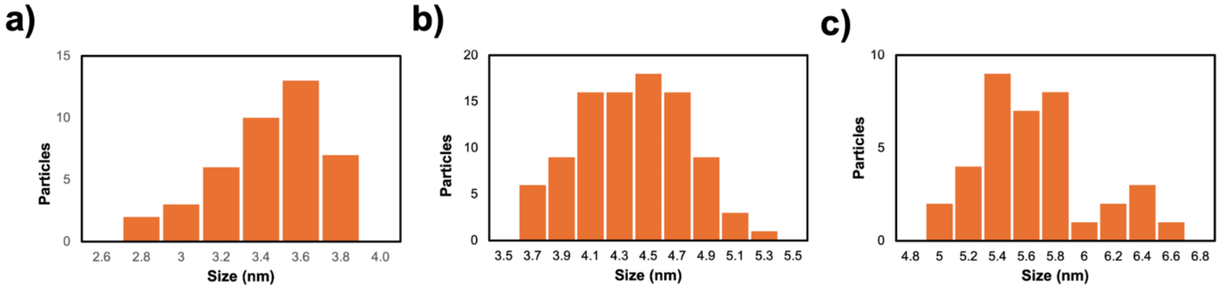

Figure S5. Size distribution histograms of **(a)** 3.4nm, **(b)** 4.3nm, and **(c)** 5.7nm QDs

Three quantum dot batches were synthesized. The sizes of the QDs were approximated using TEM to be about 3.4 nm, 4.3 nm, and 5.7 nm.

PL Results of Three 4.3 nm Samples Crosslinked with 1C and 2T:

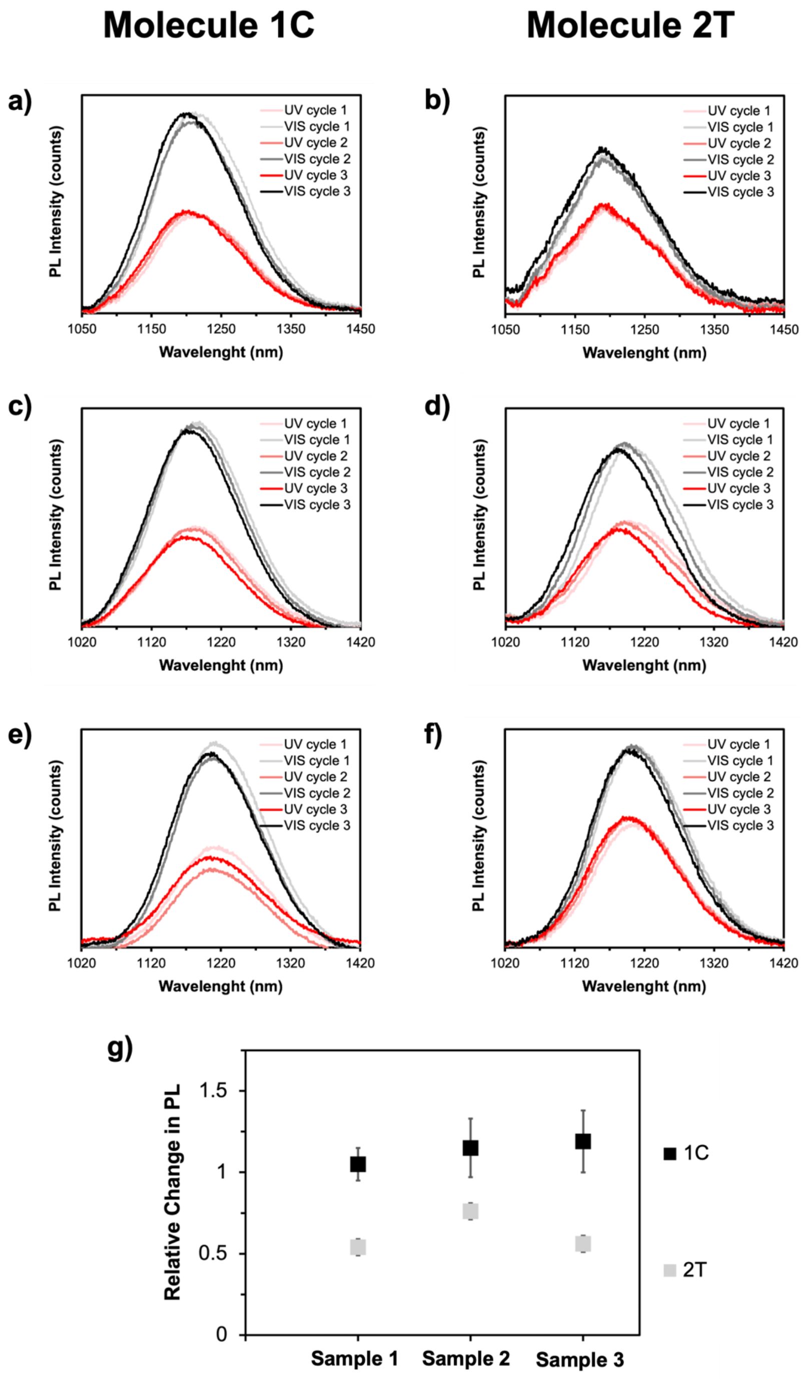

Figure S6 PL spectra of three 4.3 nm QD samples crosslinked with (**a**,**c**,**e**) **1C** and (**b**,**d**,**f**) **2T**. (**g**) Average relative change in PL of samples shows clear significant difference.

Raw Data for PL Spectra of Different Sized QDs:

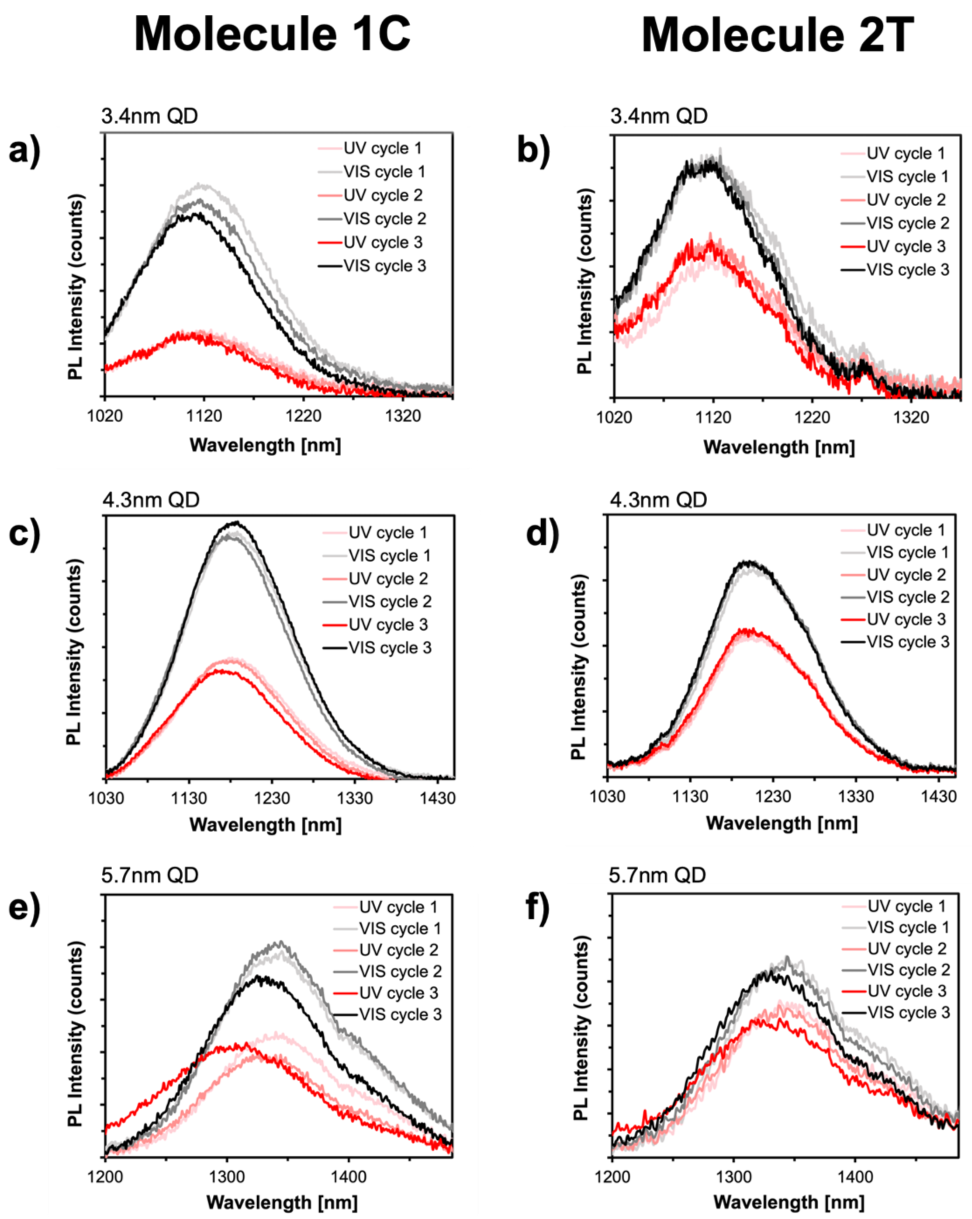

Figure S7. PL spectra of different sized QDs crosslinked with (**a**,**c**,**e**) **1C** and (**b**,**d**,**f**) **2T**

Lifetime Measurements:

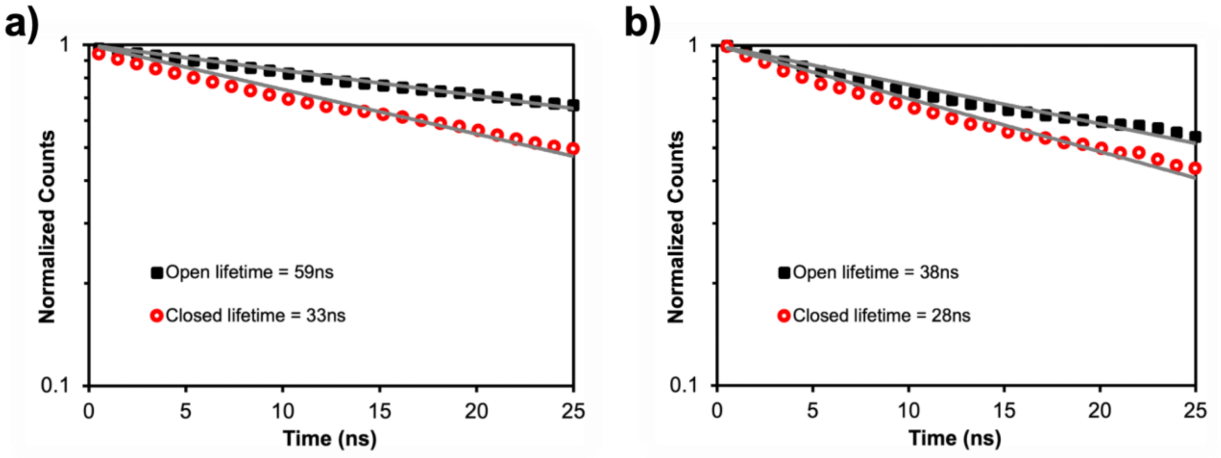

Figure S8. Excitonic lifetime measurements for 3.4nm QD films crosslinked with (**a**) **1C** and (**b**) **2T**. Fit lines for the exponentials are shown in gray.

Interestingly, the lifetime measurements obtained here can be utilized to estimate the change in tunneling rate in our system. We begin by considering the different charge decay mechanisms available in our system. These mechanisms can be grouped into three categories. Radiative recombination (which contributes to the PL), nonradiative recombination which includes all decay mechanisms other than tunneling which do not produce light, and charge tunneling which we also take to be a non-radiative decay process. Therefore, the total decay rate of the charges is given by:

$$k_{total} = k_r + k_{nr} + k_T \quad \text{(S1)}$$

Where k_{total} is the total rate of decay, $k_r$is the rate of radiative decay, k_{nr} the rate of non-radiative decay (by all pathways except for tunneling), and k_T the tunneling rate. The change in the total decay rate after configuration change of the photochromic molecules can therefore be estimated as:

$$\Delta k_{total} = k_{T,closed} + k_{T,open} \quad \text{(S2)}$$

Equation S2 therefore allows for estimates of the change in tunneling rates to be obtained utilizing the lifetime results. Using this equation Δk_{total} ~ 0.0134 nm^{-1} for **1C** and Δk_{total} ~ 0.0094 nm^{-1} for **2T**

GISAXS Results:

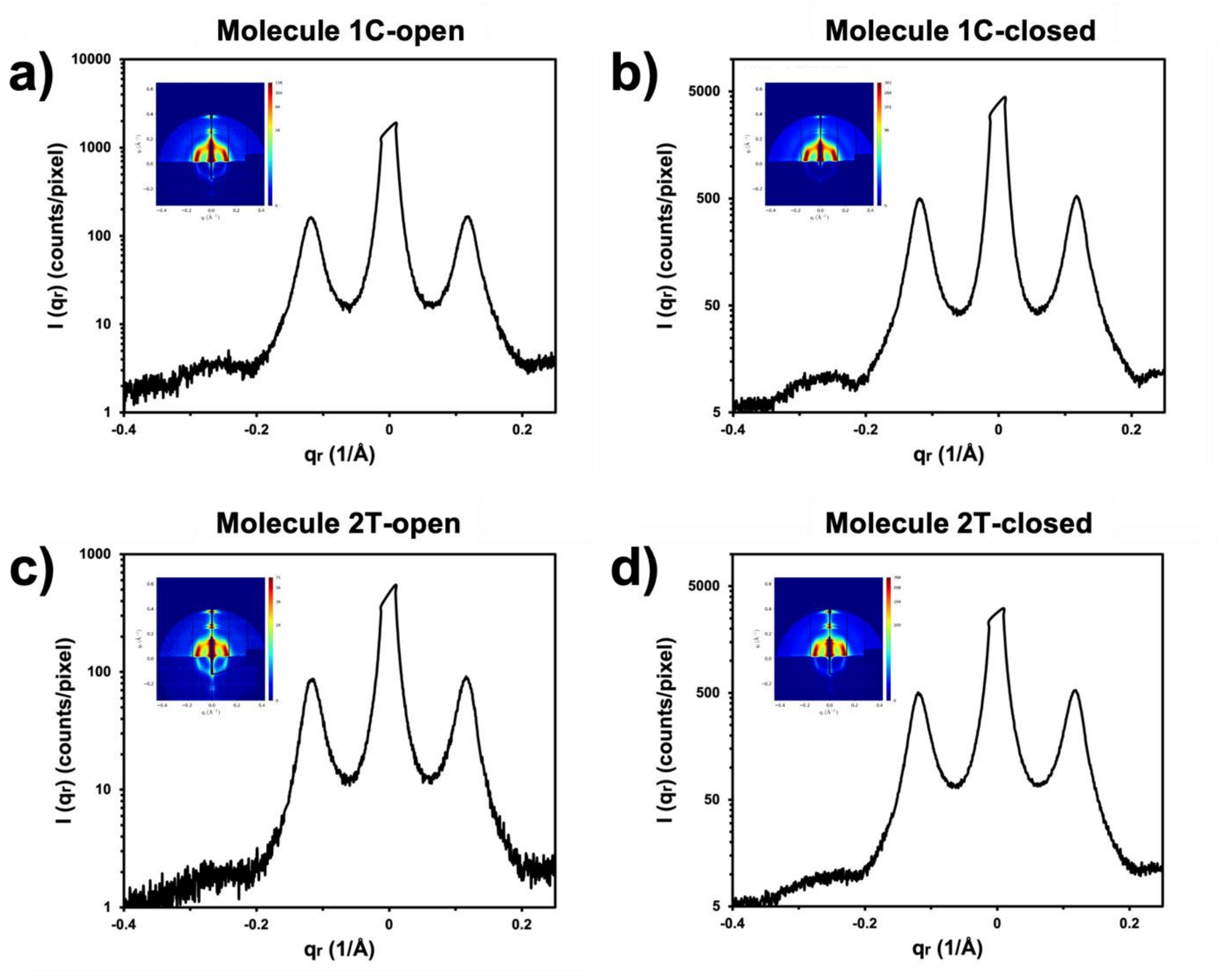

Figure S9. GISAXS measurements of molecule **1C** in the **(a)** open and **(b)** closed configurations and of molecule **2T** in the **(c)** open and **(d)** closed configurations.

Since the quantum dots used for these measurements were 4.3 nm in diameter, these results show that edge to edge distances between quantum dots were around 1 nm in length for both **1C** and **2T** and change by <0.1 nm upon configuration change.

QD size = 4.3 nm

**Connecting Ligands**	**Photochromic Molecule – "Closed" state Average Inter-QD Distance**	**Photochromic Molecule – "Open" state Average Inter-QD Distance**
Molecule 1C	53.3 Å	53.2 Å
Molecule 2T	53.3 Å	53.9 Å

Table S1. Inter-QD distance values obtained from GISAXS measurement. The displayed values are the average of the values obtained by measurements using an X-ray incident angle from 0.10 to 0.25˚.

DFT Absorbance, Orbital, and Energy Level Calculations:

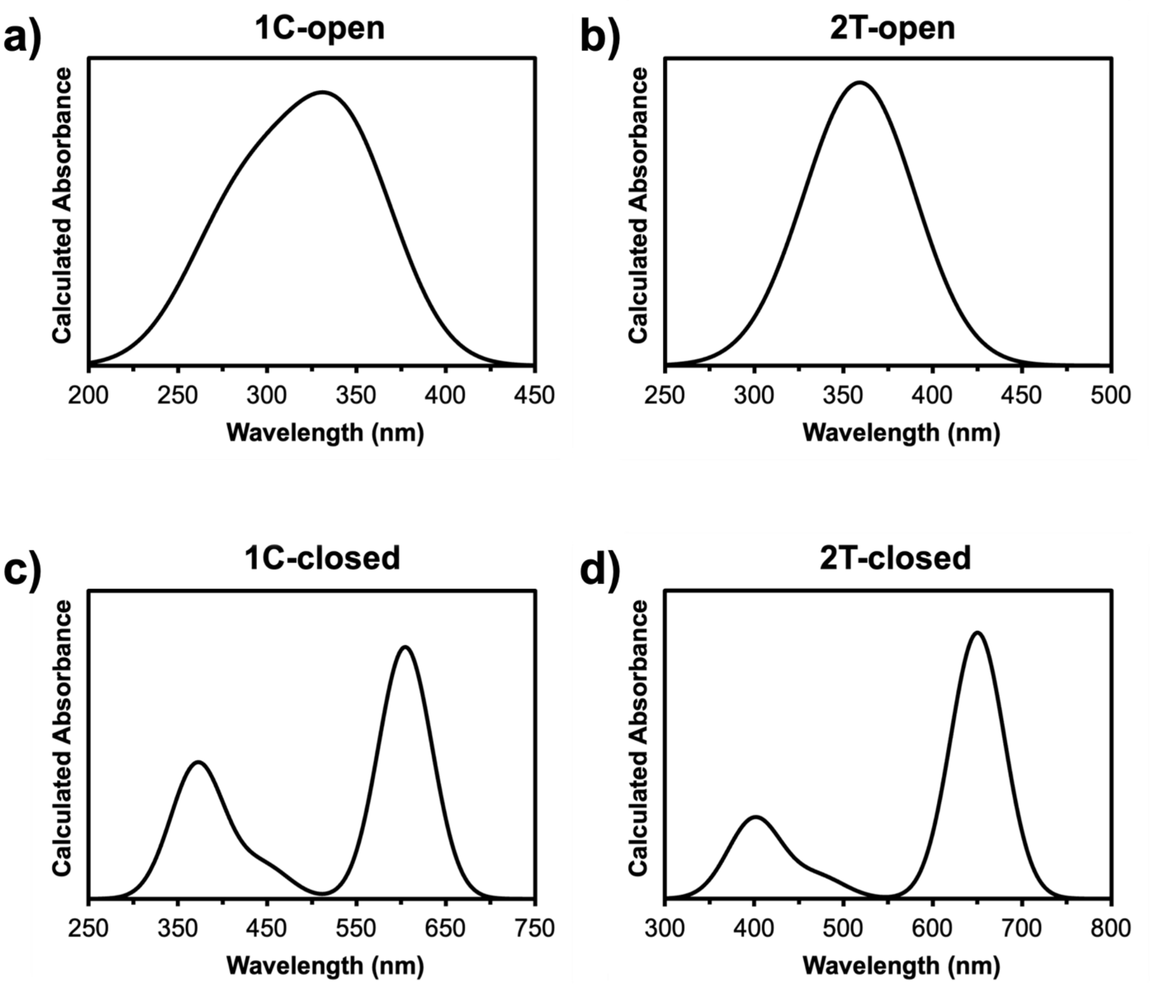

Figure S10. Calculated absorbances of ligands **1C** and **2T** in their open and closed configurations. Calculations were run using the hybrid functional B3LYP and the 6-311G basis.

Free Ligand HOMO/LUMO gaps:

Molecule	B3LYP/6-311G energy gap (eV)	PBE/PW energy gap (eV)	Experimental energy gap (eV)	PBE/PW vs B3LYP/6-311G Error
1C-closed	2.35	0.83	2.29	1.52
1C-open	4.10	2.52	4.13	1.58
2T-closed	2.20	0.68	2.07	1.52
2T-open	3.91	2.28	3.54	1.63

Table S2 HOMO/LUMO gaps for molecules **1C** and **2T** in their open and closed configurations obtained using DPV, B3LYP/6-311G functional, and the PBE functional. B3LYP/6-3111G results show good agreement with DPV results. PBE results show good agreement when accounting for an offset of ~1.55eV

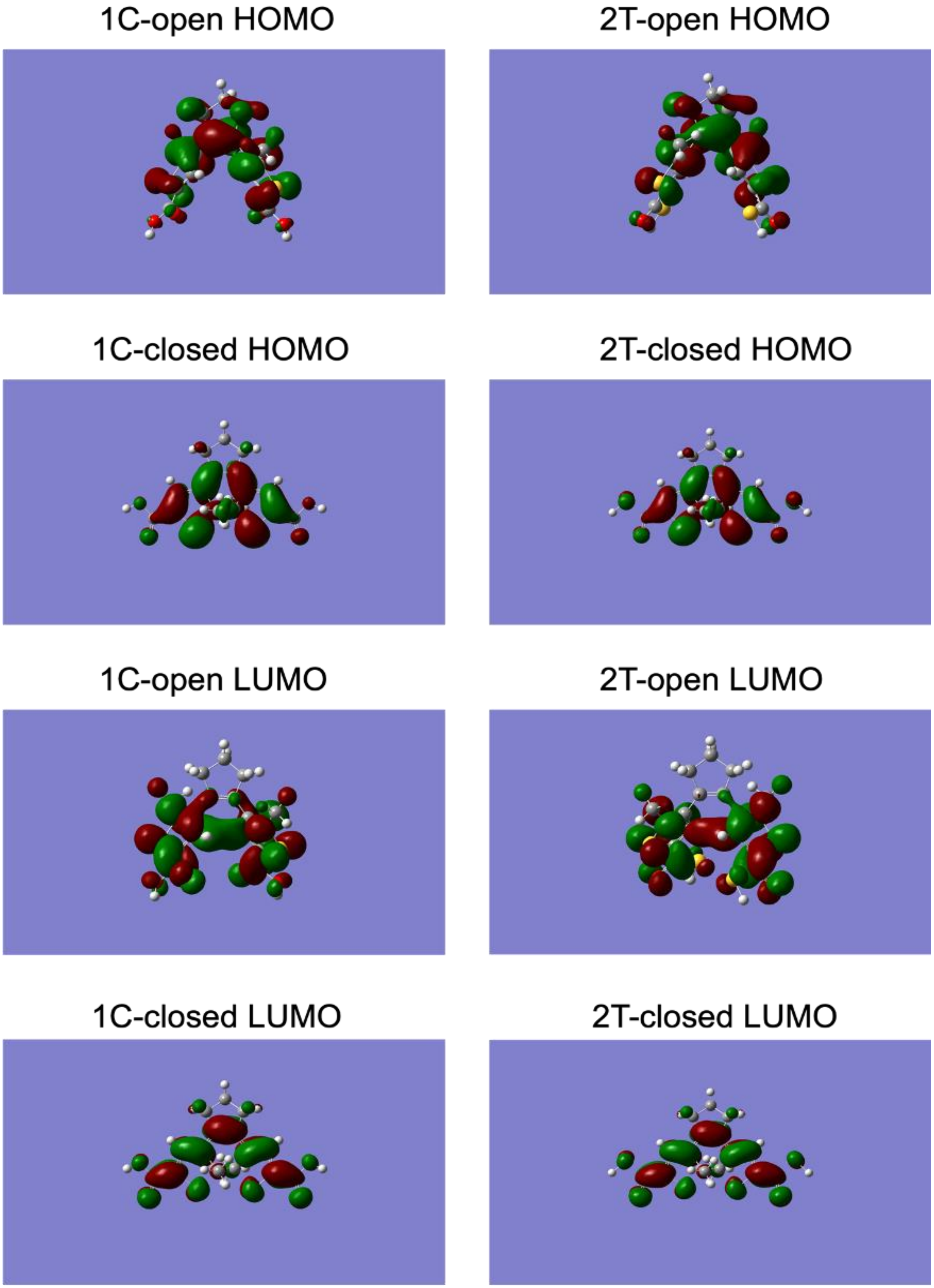

Figure S11. Orbital diagrams for ligands **1C** and **2T** in their open and closed configurations. Calculations were run using the hybrid functional B3LYP and the 6-311G basis.

Free Ligand DOS and Total Adsorbed Ligand DOS Results:

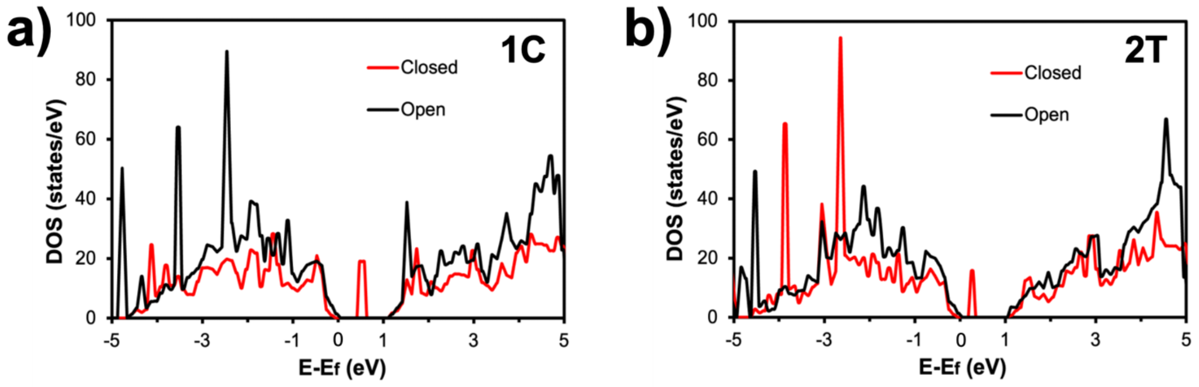

Figure S12. Calculated total DOS results for adsorbed ligands (**a**) **1C** and (**b**) **2T** using the PBE functional. Fermi energies are set to zero. Calculations were run using PBE (plane wave, PW basis set).

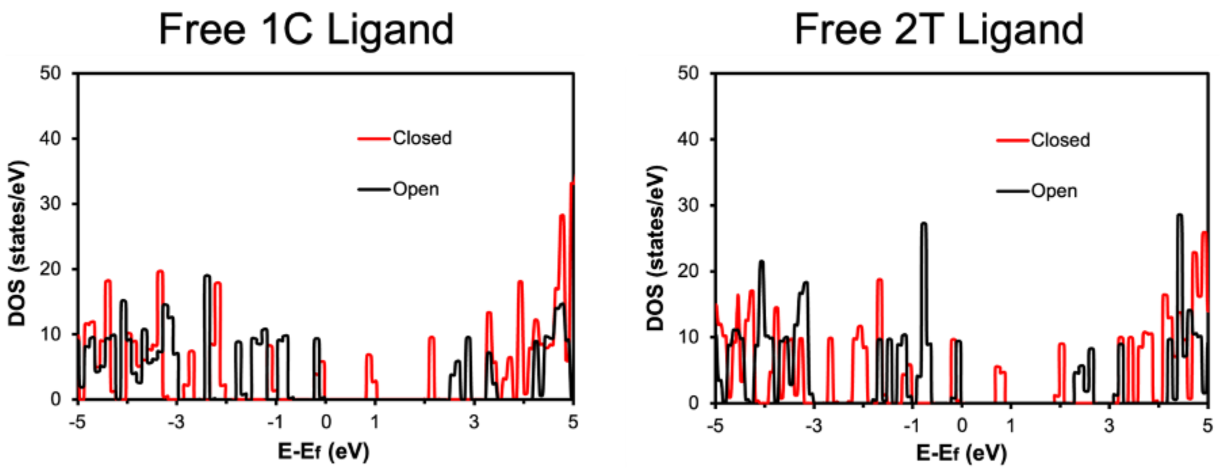

Figure S13. Calculated DOS for free ligands **1C** and **2T** using PBE functional. Fermi energies are set to zero. Calculations were run using PBE (plane wave, PW basis set).

Adsorbed vs Free Ligand HOMO/LUMO gaps:

Molecule	Adsorbed Ligand PBE/PW energy gap (eV)	Free Ligand PBE/PW energy gap (eV)
1C-closed	0.68	0.83
1C-open	2.36	2.52
2T-closed	0.55	0.68
2T-open	2.05	2.28

Table S3 Calculated HOMO/LUMO gaps for molecules **1C** and **2T** in their open and closed configurations in their free state and when adsorbed to a PbS QD surface.

Auxiliary ligand exchange mechanism in acetonitrile bath:

An auxiliary ligand exchange method was also attempted by submerging the QD-coated slides in a photochromic molecule-acetonitrile solution. 0.1 mL of a 10 mg/mL solution of QDs in tetrachloroethylene (TCE) was dispensed on cleaned and UV treated glass slides, and the slides were spin-coated at 2000 rpm for 60 s. Photochromic molecules were dissolved in a 200 mL acetonitrile bath until a concentration of 0.1 mM was achieved. The slides were carefully deposited in the acetonitrile bath. The bath was stirred using a magnetic stir bar and sealed to minimize solvent evaporation. After the target time elapsed, the slides were extracted from the bath, dried with air, and sealed with epoxy. **1C** and **2T** slides were found to reach an asymptotically maximal PL switching amount after roughly 30 minutes in the bath. Results mirrored those in **Fig. 3**, with **1C** samples displaying greater relative change in PL than the **2T** samples.

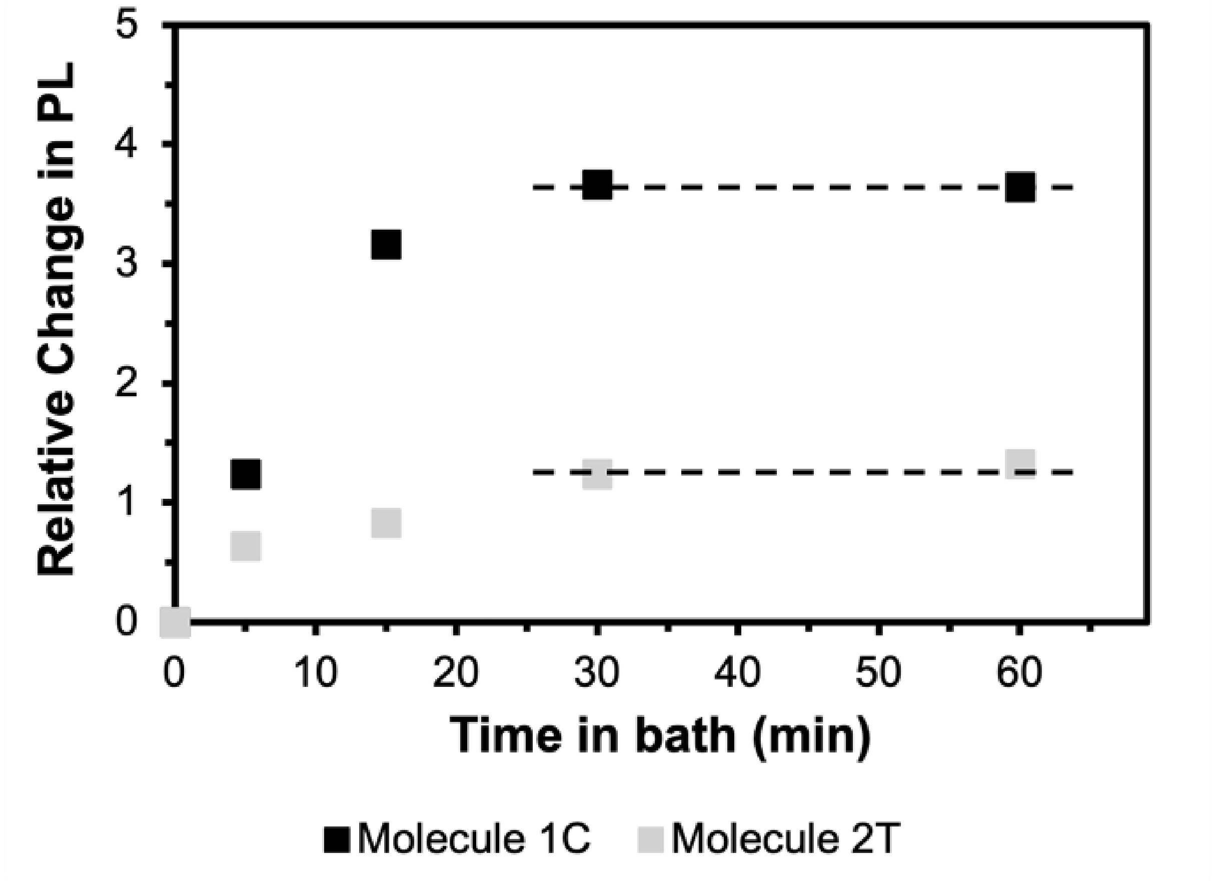

Figure S14. Relative change in PL of 3.4nm QD slides crosslinked with **1C** and **2T** after different times in the acetonitrile bath.

References [SI]:

(1) Lucas, L. N.; De Jong, J. J. d.; Esch, J. H. v.; Kellogg, R. M.; Feringa, B. L. Syntheses of dithienylcyclopentene optical molecular switches. *European Journal of Organic Chemistry* **2003**, *2003* (1), 155-166.
(2) Lucas, L. N.; van Esch, J.; Kellogg, R. M.; Feringa, B. L. A new class of photochromic 1, 2-diarylethenes; synthesis and switching properties of bis (3-thienyl) cyclopentenes. *Chemical Communications* **1998**, (21), 2313-2314.
(3) Yoon, L. U.; Adhikari, S. B.; Sarabamoun, E. S.; Bietsch, J. M.; Tsai, E. H. R.; Wang, G.; Choi, J. J. Exciton dissociation in quantum dots connected with photochromic molecule bridges. *Journal of Materials Chemistry C* **2021**, *9*(44), 16006-16013, 10.1039/D1TC04451F. DOI: 10.1039/D1TC04451F.
(4) Sarabamoun, E. S.; Bietsch, J. M.; Aryal, P.; Reid, A. G.; Curran, M.; Johnson, G.; Tsai, E. H. R.; Machan, C. W.; Wang, G.; Choi, J. J. Photoluminescence switching in quantum dots connected with fluorinated and hydrogenated photochromic molecules. *RSC Advances* **2024**, *14* (1), 424-432, 10.1039/D3RA07539G. DOI: 10.1039/D3RA07539G.
